# FTO Gene Variant and Risk of Overweight and Obesity among Children and Adolescents: a Systematic Review and Meta-Analysis

**DOI:** 10.1371/journal.pone.0082133

**Published:** 2013-11-22

**Authors:** Chibo Liu, Sihua Mou, Yangqun Cai

**Affiliations:** Department of Clinical Laboratory, Taizhou Municipal Hospital, Taizhou, Zhejiang, China; Harvard Medical School, United States of America

## Abstract

**Objective:**

The fat mass and obesity associated gene (FTO) polymorphisms have been implicated in the susceptibility of overweight/obesity in children and adolescents. However, the results have been inconsistent. In this study, we performed a meta-analysis to clarify the association of FTO gene polymorphisms with overweight/obesity risk among children and adolescents.

**Methods:**

PubMed and Embase were used to search for eligible published literatures. Pooled odds ratios (ORs) with 95% confidence intervals (CIs) were calculated using random- or fixed-effect models.

**Results:**

A total of 21 articles containing 23 studies (11208cases and 35015controls) were included in our analysis. The results indicated that variant in FTO gene was significantly associated with increased risk of overweight/obesity in children and adolescents (OR=1.35; 95%CI: 1.27-1.44; *P*<0.001). The overall pooled ORs for risk obesity and overweight were 1.34 (95%CI: 1.21-1.48) and 1.35 (95%CI: 1.25-1.47), respectively. Subgroup analyses also showed similar trends in most subgroups of adjustment for covariates and unadjustment, different ethnicities (Caucasians, Asians, and Amerindians), and each of three investigated polymorphisms (rs9939609, rs1421085, and rs1558902).

**Conclusions:**

The present meta-analysis suggested a positive association between FTO gene polymorphism and overweight/obesity risk among children and adolescents. Further prospective studies should be recommended to confirm the observed association, and underlying mechanism should be investigated to clarify the association of FTO gene polymorphism with overweight/obesity.

## Introduction

Obesity, which is associated with an increased risk of many chronic diseases, including type-2 diabetes, cardiovascular diseases and cancer, has become a major health problem worldwide [1]. More than 400 million people are obese across the world and the number will reach 700 million by 2015, according to the World Health Organization. Obesity is also increasingly prevalent among children and adolescents. Approximately 38.1% and 16.9% of children and adolescents in US are overweight and obesity, respectively[2]. If obese occurs during childhood, especially at adolescence, it is more likely to remain obese when an individual become adult as well as increases the risk of adult morbidity and mortality [3-5]. Therefore, overweight/obesity in childhood not only influences health and well-being during childhood and adolescence, but also has potential long-term health consequences for later adult chronic diseases.

Obesity is a complex multifactor disease that is affected by environmental risk factors and biological ones (including monogenic variations and common genetic variants)[6]. Approximately 40%-70% adult obesity is attributed to genetic factors[5]. Recently, many single nucleotide polymorphisms (SNPs) were identified by genome-wide association studies (GWASs) in adults and children with respect to the pathogenesis of obesity, including *FTO*, *TMEM18*, *GNPDA2*, *INSIG2*, *MC4R*, *NEGR1*, *1q25*, *BDNF* and *KCTD15*[7]. *FTO*, a gene located in chromosome region 16q12.2, was the first identified gene for common obesity[8]. Subsequently, the association of *FTO* variants with BMI and obesity has been investigated in different ethnic populations. A previous meta-analysis by Peng et al. [9] indicated the association between FTO gene polymorphisms and obesity risk. However, the paper by Peng et al. was mainly based on adult populations and only limited studies on children populations were included. Indeed, the results are still inconsistent in children populations. In this study, a meta-analysis was performed to clarify the association of common genetic variants in *FTO* with overweight/obesity risk in children and adolescents. 

## Materials and Methods

### Literature and search strategy

We searched the literature databases including PubMed and Embase from 2007 to 2013 since rs9939069 polymorphism in FTO gene and its association with obesity was first reported in 2007. The search strategy to identify all possible studies involved the use of the following keywords: (fat mass and obesity associated gene OR *FTO*) and (polymorphism OR variant OR variation OR genotype) and (children OR adolescents) and (obesity OR overweight). The publication language was restricted to English. The reference lists of retrieved articles were hand-searched. If more than one article were published using the same case series, only the study with largest sample size was included. The literature search was updated on 20 June 2013.

### Inclusion criteria and data extraction

The included studies fulfilled the following inclusion criteria: (1) an original report evaluating the association of any of the *FTO* polymorphisms with overweight/obesity risk; (2) using case-control, cross-sectional, or cohort design; (3) providing an odds ratio (OR) or relative risk (RR) with 95% confidence interval (CI) under an additive model or sufficient raw data to calculate it; and (4) participants should be children and/or adolescents. The following information was extracted from each study: (1) name of the first author; (2) year of publication; (3) origin of country; (4) ethnicity of studied population; (5) number of cases and controls; (6) mean age and body mass index (BMI) of subjects; (7) OR or RR with 95% CI under an additive model; (8) adjustment for covariates; (9) studied SNPs; (10) genotype distribution in cases and controls; and (11) *P* value for Hardy-Weinberg equilibrium (HWE) test in controls. Two authors independently assessed the articles for compliance with the inclusion/exclusion criteria, and resolved discrepancies by group discussion until reaching a consistent decision.

### Statistical analysis

The association between *FTO* polymorphism and overweight/obesity risk was estimated by calculating pooled OR with 95%CI under an additive model. The significance of pooled OR was determined by Z test (*P*<0.05 was considered statistically significant). The between-study heterogeneity was evaluated by Q statistic and *I* ^2^ index[10]. A random-(DerSimonian-Laird method[11]) or fixed-(Mantel-Haenszel method[12]) effects model was used to calculate pooled OR in the presence (*P* <0.10) or absence (*P*>0.10) of heterogeneity, respectively. Subgroup analyses were conducted by ethnicity, adjustment, *FTO* SNP, and category of cases. Sensitivity analysis, after removing one study at a time, was performed to evaluate the stability of the results. Begg’s funnel plot[13], a scatter plot of effect against a measure of study size, was generated as visual aid to detect bias or systematic heterogeneity. Publication bias was assessed by Begg’s test[13]and Egger’s test[14](*P*<0.05 was considered statistically significant). Data analysis was performed using STATA version 11(StataCorp LP, College Station, TX, USA).

## Results

### Characteristics of the included studies

**Figure 1:**
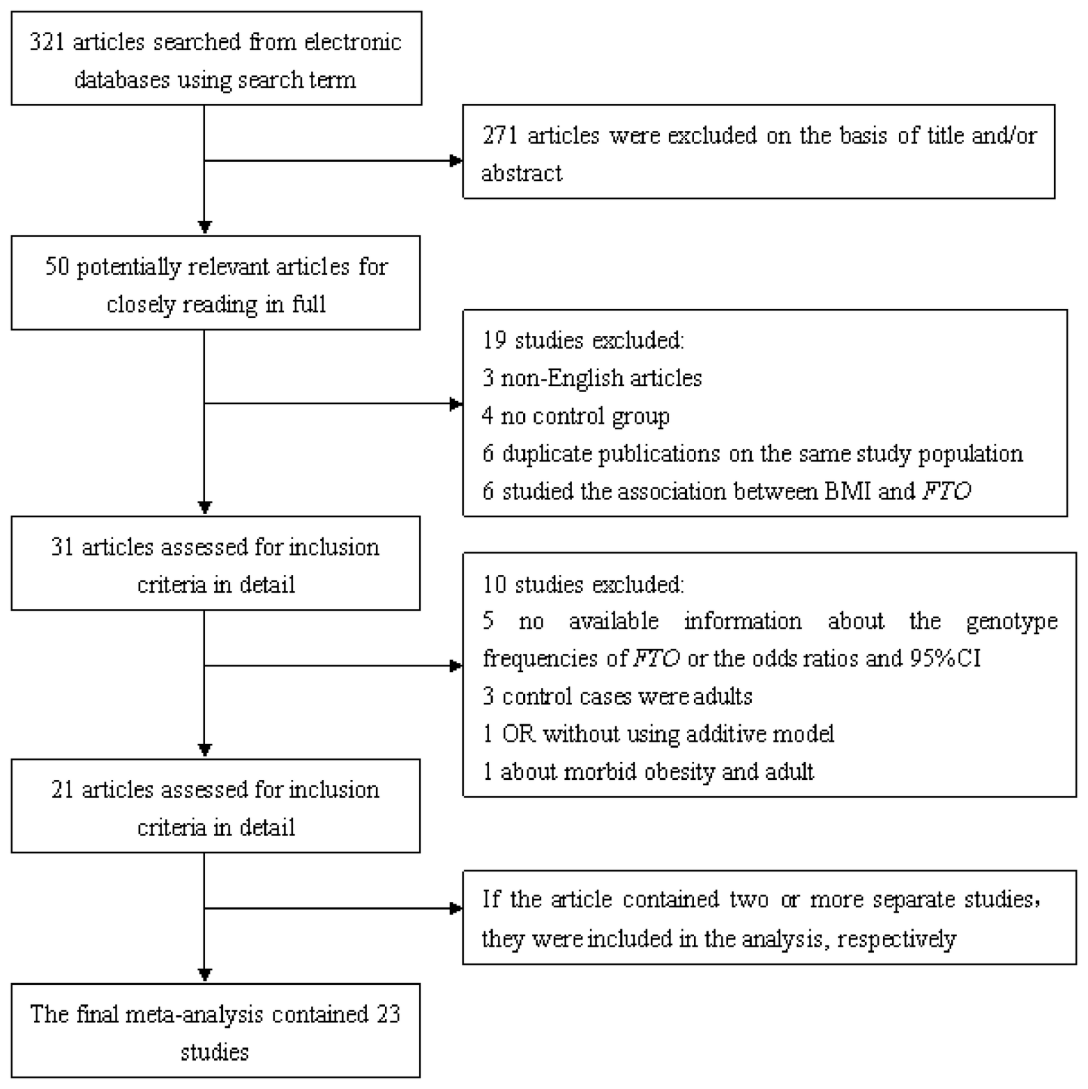
Flow chart of article selection for meta-analysis.

Figure 1 provides the detailed process of articles’ selection. A total of 321 articles were identified from the primary literature search. After review of the titles and abstracts, 271 were excluded because of obvious irrelevance. 50 potentially relevant articles remained for further full-text evaluation. Of these, 3 articles were excluded because of non-English articles, 4 articles were excluded as they did not have control groups, 6 articles were excluded due to duplicate publications, and 6 articles were excluded since they assessed the association between BMI and FTO gene polymorphism. Furthermore, 5 papers which did not provide available information about the genotype frequencies of *FTO* or the ORs and 95%CIs were also excluded [15-19]. 3 papers were excluded as the controls were adults [20-22]. One report without using additive model to calculate OR was excluded[23]. And one paper was also excluded for studying early onset and morbid obesity of adult when they were before 14 years old[24]. Consequently, 21 eligible articles met the inclusion criteria. If the article contained two or more studies, they were included as separate study in the data analysis. As a result, 21 articles containing 23 studies (11208 cases and 35015 controls) were included in the final meta-analysis.

The genotypes in the controls of all included studies were in Hardy-Weinberg equilibrium, except for two those were unknown [25,26] and two studies stated P for HWE was >0.01 [41,43]. In addition, there were 14 studies for rs9939609 [8,25,27-37], 4 studies for rs1421085 [38-41], 3 studies for rs1558902[26,42,43], 2 studies for rs8050136 [44]. Owing to the high linkage disequilibrium with the other *FTO* SNPs (*r* ^2^>0.85), rs9939609 was considered as the surrogate polymorphism. The ethnicities varied across studies: 16 were on Caucasian population [8,25-28,31,33,35-40,43,44], 4 were on Asian population[29,30,32,42], 2 was on Amerindian population [34,41], and 1 was on African population [44]. As most ORs or RRs and 95%CIs of studies were generated under an additive genetic model, the final pooled estimate was under this model. The characteristics of the included studies are shown in Table 1.

**Table 1:** Characteristics of studies included in meta-analysis.

Study	Year	Country	Ethnicity	*FTO* SNP	Characteristics of cases **^*a*^**	Sample size		Mean age(years)		Mean BMI(kg/m^2^)		OR	95%CI	*P*_HWE_ **^*b*^**	Adjustment
						Cases	Controls	Cases	Controls	Cases	Controls				
Frayling_a[8]	2007	UK	Caucasian	rs9939609	Overweight	1304	3706	11		18.76		1.27	1.16-1.39	0.658	Gender
Frayling_b[8]	2007	Finland	Caucasian	rs9939609	Overweight	308	3895	14		19.23		1.15	0.98-1.36	0.195	Gender
Dina[38]	2007	Germany	Caucasian	rs1421085	Overweight	283	699	11.6	11.7	NA	NA	1.69	1.38-2.06	0.87	NA
Wardle[27]	2008	UK	Caucasian	rs9939609	Obese	926	4022	10.7	10	NA	NA	1.76	1.59-1.94	0.497	NA
Jacobsson[28]	2008	Sweden	Caucasian	rs9939609	Obese	450	510	12.6	17.1	35.4	21.1	1.254	1.047-1.502	0.7567	NA
Grant_a[44]	2008	America	Caucasian	rs8050136	Obese	418	2270	NA	NA	NA	NA	1.266	1.088-1.471	equilibrium	NA
Grant_b[44]	2008	America	African	rs8050136	Obese	578	1424	NA	NA	NA	NA	1.051	0.914-1.207	equilibrium	NA
Meyre [39]	2009	Germany	Caucasian	rs1421085	Obese	370	710	11.8	11.8	29.72	18.15	1.498	1.253-1.791	0.081	NA
Cauchi[40]	2009	Finland	Caucasian	rs1421085	Obese	247	4023	16		21.29		1.44	1.19-1.75	0.222	Age, gender
Lee[29]	2010	Korea	Asian	rs9939609	Overweight	140	571	8.05	8.16	20.46	15.94	1.53	1.06-2.22	0.533	Age, gender
Xi[30]	2010	China	Asian	rs9939609	Obese	1229	2274	11.8	12.7	26.5	19.4	1.29	1.11-1.5	0.146	Age, gender
Scherag[43]	2010	Germany	Caucasian	rs1558902	Overweight	711	1803	10.71	NA	27.49	NA	1.35	1.19-1.52	>0.01	Age, gender
Mangge[31]	2011	Austria	Caucasian	rs9939609	Obese	268	103	12.5	14	30.1	20	1.341	0.97-1.854	0.26	NA
Okuda[42]	2011	Japan	Asian	rs1558902	Overweight	130	133	NA	NA	23.5	17.6	2.2	1.43-3.38	0.613	Gender
Dwivedi[32]	2012	India	Asian	rs9939609	Overweight	848	2147	13.45	13.51	26.32	17.97	1.21	1.07-1.37	0.817	Age, gender
Luczynski[33]	2012	Poland	Caucasian	rs9939609	Overweight	334	634	14.01		NA	NA	1.433	1.187-1.73	0.886	NA
Riffo[34]	2012	Chile	Amerindian	rs9939609	Obese	238	136	8.55	8.42	NA	NA	1.422	1.068-1.868	0.06	NA
Moleres[36]	2012	Spain	Caucasian	rs9939609	Obese	208	146	11.6	11.5	27.4	19	1.419	1.048-1.92	0.329	NA
Lauria[35]	2012	Italy	Caucasian	rs9939609	Overweight	808	3597	6.06		16.37		1.41	1.12-1.77	0.378	Age, gender country of origin
Almén[25]	2012	Sweden	Caucasian	rs9939609	Obese	524	527	12.7	17	NA	NA	1.25	1.05-1.48	NA	Gender
Ntalla[26]	2013	Greece	Caucasian	rs1558902	Overweight	218	489	13.42		21.3		1.33	1.06-1.67	NA	Age, gender
Albuquerque[37]	2013	Portugal	Caucasian	rs9939609	Obese	154	247	9	8.6	23.8	16.1	1.427	1.071-1.9	0.732	Age, gender
Mejía-Benítez[41]	2013	Mexico	Amerindian	rs1421085	Obese	514	949	9.5	9.5	25	17.7	1.13	0.93-1.38	>0.01	Age, gender

NA no available, OR odds ratio, CI confidence interval, SNP single nucleotide polymorphism

***^a^*** The overweight group comprised overweight and obese children and adolescents ***^b^*** *P* value for Hardy–Weinberg equilibrium test (HWE) in controls

### Quantitative assessment

**Figure 2:**
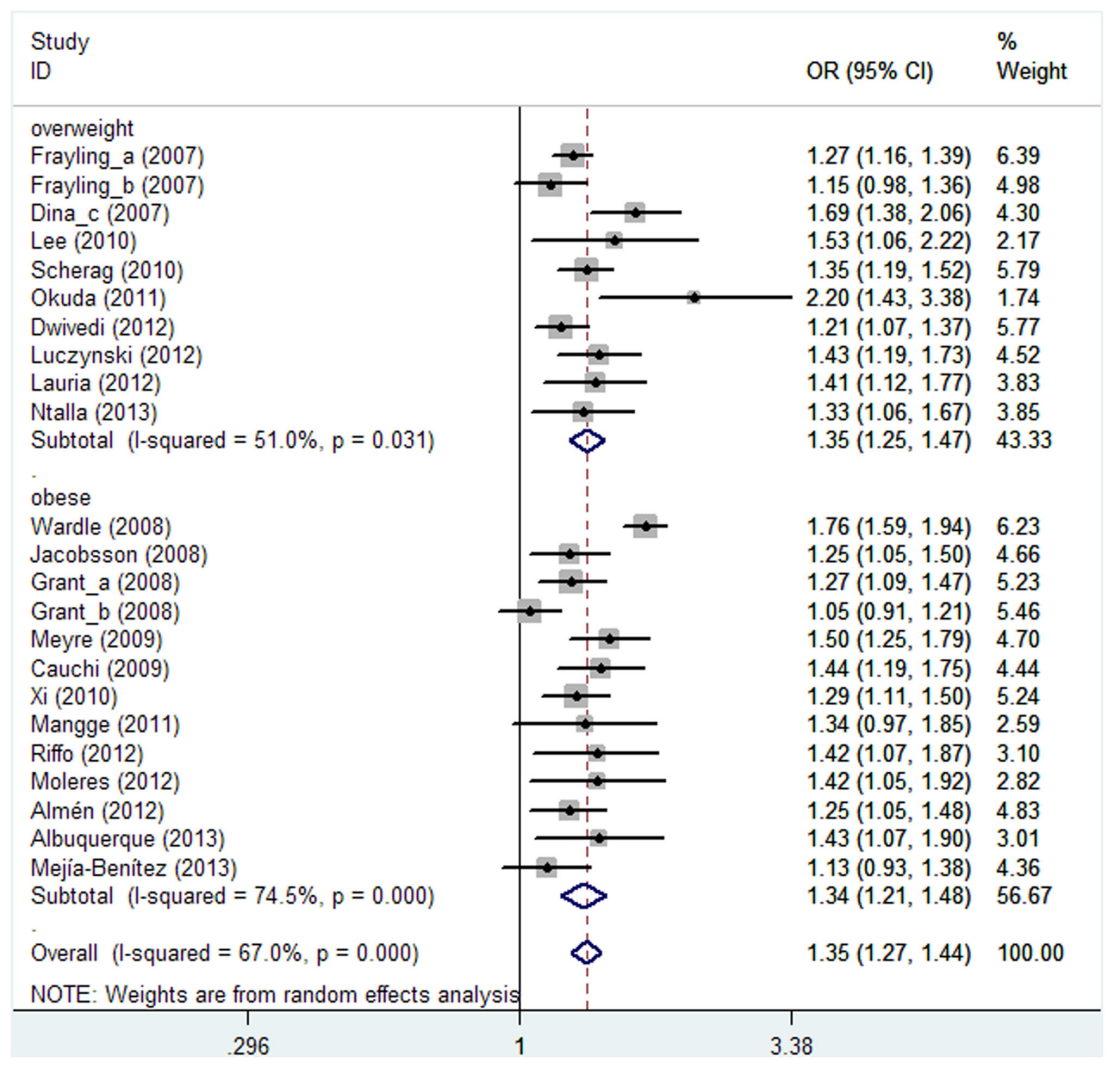
Forest plot of meta-analysis of the association between rs9939609 (or its proxy) and overweight/obesity among children and adolescents under an additive model.

Regarding to the association of *FTO* rs9939609 (or its proxy) with risk of overweight/obesity in children and adolescents, there was evidence of heterogeneity between studies (*P*<0.001, *I* ^2^=67.0%). Therefore, the random effect model was used. The result showed a statistically significant association between rs9939609 and overweight/obesity, with an overall OR of 1.35 (95%CI=1.27-1.44; Figure 2). Since the underlying etiology of overweight and obesity may be different and genetic variation may have different effects on them, further analysis based on categories of cases were performed. The overall pooled OR of risk obesity was 1.34(95%CI: 1.21-1.48, Figure 2), with evidence of heterogeneity between studies (*I* ^2^=74.5%; *P*<0.001). The overall pooled OR of risk overweight was 1.35(95%CI: 1.25-1.47, Figure 2), with moderate of heterogeneity between studies (*I* ^2^=51.0%, *P*=0.031). In the stratified analysis by adjustment, the pooled summary OR with the adjusted data showed significant association (OR=1.29; 95%CI: 1.23-1.35; *I* ^2^=17.4%; *P* for heterogeneity=0.268), and the overall analysis without adjusted data also showed the similar result (OR=1.40; 95%CI: 1.23-1.59; *I* ^2^=79.2%; *P* for heterogeneity<0.001). Further subgroup analysis stratified by ethnicity showed significant association between rs9939609 and overweight/obesity risk in Caucasians (OR=1.38; 95%CI: 1.29-1.49; *I* ^2^=62.3%; *P* for heterogeneity<0.001), in Asians (OR=1.38; 95%CI: 1.16-1.65; *I* ^2^=61.4%; *P* for heterogeneity=0.051), and in Amerindians (OR=1.22; 95%CI=1.04-1.43; *I* ^2^=42.3%; *P* for heterogeneity=0.188), but not in Africans (OR=1.05; 95%CI: 0.91-1.21) . In addition, we also performed a stratified analysis according to different polymorphisms. Except for rs8050136 (OR=1.15, 95%CI: 0.96-1.38), all the polymorphisms showed a strong correlation with increased risk of being overweight/obese (rs9939609: OR=1.35, 95%CI: 1.24-1.47; rs1421085: OR=1.43, 95%CI: 1.21-1.67; rs1558902: OR=1.46, 95%CI: 1.19-1.79). The results of subgroup analyses were showed in Table 2.

**Table 2:** Meta-analyses of association between *FTO* polymorphisms and overweight/obesity risk under an additive model

Contrasts	No.of study (cases/controls)	Statistical model	OR	95%CI	*P*_z_ **^*a*^**	*I2*	*P*_H_ **^*b*^**
All studies	23(11208/35015)	Random	1.35	1.27-1.44	<0.001	67	<0.001
Categories of cases							
Obesity	13(6124/17341)	Random	1.34	1.21-1.48	<0.001	74.5	<0.001
Overweight	10(5084/17674)	Random	1.35	1.25-1.47	<0.001	51.0	0.031
Ethnicity							
Caucasians	16(7531/27381)	Random	1.38	1.29-1.49	<0.001	62.3	<0.001
Asians	4(2347/5125)	Random	1.38	1.16-1.65	<0.001	61.4	0.051
Amerindians	2(752/1085)	Fixed	1.22	1.04-1.43	0.016	42.3	0.188
Africans	1(578/1424)		1.05	0.91-1.21	0.483		
Adjustment							
Yes	13(7135/24361)	Fixed	1.29	1.23-1.35	<0.001	17.4	0.268
No	10(4073/10654)	Random	1.40	1.23-1.59	<0.001	79.2	<0.001
Polymorphisms							
rs9939609	14(7739/22515)	Random	1.35	1.24-1.47	<0.001	66.4	<0.001
rs1421085	4(1414/6381)	Random	1.43	1.21-1.67	<0.001	64.3	0.038
rs1558902	3(1059/2425)	Random	1.46	1.19-1.79	<0.001	57.8	0.094
rs8050136	2(996/3694)	Random	1.15	0.96-1.38	0.131	68.4	0.075

*a* *P* value for Z test

*b* *P* value based on Q test for between-study heterogeneity

### Sensitivity analysis

To test the sensitivity of the meta-analysis, we excluded each study at one time. The corresponding pooled ORs were not materially altered. After excluding the two studies where the HWE of the genotypes in controls were unknown, and two studies where *P* for HWE was more than 0.01, the results did not substantially change, with ORs ranging from 1.30(95%CI:1.25-1.35) to 1.38(95%CI:1.33-1.44). Thus, the significant association for rs9939609 with overweight/obesity risk was statistically robust by sensitivity analysis.

### Potential publication bias

**Figure 3:**
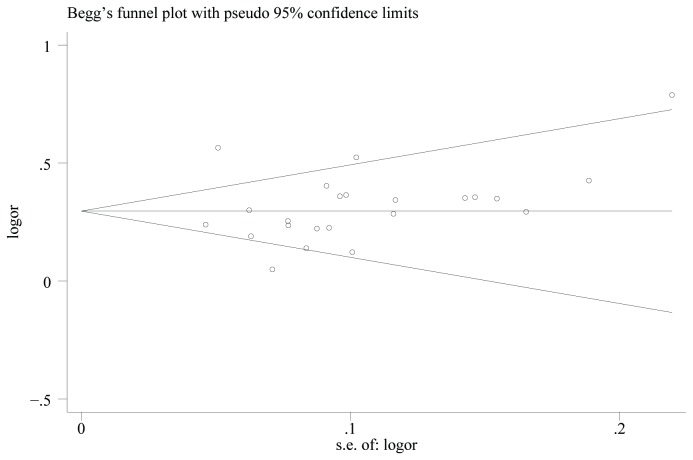
Begg’s funnel plot for publication bias in an additive model.

The Begg’s funnel plot did not reveal any evidence of obvious asymmetry (Figure 3), and both Egger’s test and Begg’s test were not significant (Begg’s test: *P*=0.064; Egger’s test: *P*=0.637). Therefore, no publication bias was detected in this mate-analysis. 

## Discussion

In this study, our meta-analysis confirmed that variant in FTO gene was significantly associated with increased risk of overweight/obesity in children and adolescents. The stability of sensitivity analysis and no publication bias confirmed the positive association. Further subgroup analyses indicated the similar trends among subgroups of overweight and obesity, data with and without adjustment, different ethnicities (Caucasians, Asians, and Amerindians ), and each of three polymorphisms investigated (rs9939609, rs1421085, rs1558902).

Between-study heterogeneity is usually common in meta-analysis of genetic association study[45]. As the evidence of heterogeneity in our study, subgroup analyses based on ethnicity, categories of cases, adjustment, and types of polymorphisms were used to assess the potential source and impact of heterogeneity. Nevertheless, there were still between-study heterogeneities in some subgroups, suggesting other unknown influence factors.

The underlying biological mechanism on how the variant in *FTO* contributes to the increased risk of obesity is largely unknown[7]. For animal experiments, there is a demonstration that *FTO* plays an important role in energy homeostasis, metabolism and adipogenesis [46,47]. To elucidate the way in which the *FTO* variant affects fat mass may help us to understand the pathogenesis of obesity[33]. FTO gene contributes to weight gain mainly by increasing in energy intake[32] and diminishing satiety sensation[27] . Meanwhile, mutation of *FTO* was influenced by higher fatty acid intake[36],. Some reports have suggested that FTO gene products influence the regulation of food intake, as children carrying the risk allele tend to choose higher energy and more fat food[48,49]. Besides, carriers with high-risk allele for FTO gene is more resistant to the insulin effects than the ones with non-risk allele of genetic variant[50]. The FTO gene is expressed in many tissues, including human hypothalamus, pituitary and adrenal glands[38]. Regarding slight change of TSH (Thyroid Stimulating Hormone) levels associating with weight gain and fat mass[51], influence of *FTO* variants on pituitary function has a strong association with TSH levels, for both of them are expressed or produced in pituitary gland[32]. However, *FTO* genetic effects do not begin to influence early onset obesity before the age of 7 years[52]. Despite genotyping of *FTO* in childhood may be effective in identifying individuals genetically predisposed to obesity, the lifestyle factors also should be considered [35]. Further in-depth researches are needed to explore the mechanism by which FTO associated with overweight/obesity.

The result of pooled effect for rs9939609 genetic variant of FTO gene with overweight/obesity risk in our meta-analysis (OR=1.35; 95%CI: 1.27-1.44) was similar with previous overall result of rs9939609 by Peng et al. (OR=1.31; 95%CI: 1.26-1.36)[9], but was also similar with that of rs17782313 (or its proxy) near the *MC4R* gene, another obesity susceptibility gene, in children reported by Xi et al. (OR=1.26; 95%CI: 1.19-1.33)[53]. However, we did not identify the association between the FTO gene rs8050136 and overweight/obesity risk (OR=1.15; 95%CI: 0.96-1.38), in spite of a significant positive association found by Peng et al. (OR=1.25; 95%CI: 1.13-1.38)[9]. The overall analysis also showed no evidence of association between rs9939609 (or its proxy) and overweight/obesity risk in African children and adolescents. The result might be explained by some other different biological effects. Aerobic capacity of African American might be lower than white children[54]. Moreover, fasting insulin and acute insulin response were significantly higher in African American, while insulin sensitivity was significantly lower[54]. Further investigations on rs8050136 and African should be pursued to determine the association of FTO gene with overweight/obesity.

Despite the many strengths compared to individual studies, there are several limitation in our meta-analysis. First, some potential confounding factors were not controlled for because our pooled estimate was based primarily on unadjusted estimates and CIs. Second, different criteria of obesity and overweight were included in our meta-analysis. Therefore, these results should be interpreted with caution. Trying to overcome this drawback, we performed subgroup analysis by ethnicity, which indirectly reflected the differences in obesity and overweight criteria as the cut-offs were similar in the same ethnic group. Third, a number of obesity-related clinical and biochemical parameters were not estimated in this meta-analysis. Forth, owing to insufficient original data from the included studies, the effects of gene-gene and gene-environment interactions were not assessed. 

In summary, our meta-analysis results indicated that rs9939609 polymorphism in the FTO gene was significantly associated with overweight/obesity risk in children and adolescents. However, further studies considering gene-gene and gene-environment interaction should be conducted to investigate the association. Furthermore, better estimates of the risk would be obtained in future prospective cohort studies, as well as more mechanism researches are also needed to clarify the association of FTO gene with overweight/obesity. 

## Supporting Information

Checklist S1 **PRISMA checklist.**
(DOC)
